# Ly6G^+^ Neutrophils and Interleukin-17 Are Essential in Protection against Rodent Malaria Caused by *Plasmodium berghei* ANKA

**DOI:** 10.34133/research.0559

**Published:** 2024-12-19

**Authors:** Ziwei Su, Qilong Li, Yiwei Zhang, Tong Liu, Kunying Lv, Anni Feng, Yixin Yang, Yanxin Zhang, Zhiming Wei, Xiaoyu Sang, Ying Feng, Ran Chen, Ning Jiang, Qijun Chen

**Affiliations:** ^1^ Key Laboratory of Livestock Infectious Diseases, Ministry of Education, and Key Laboratory of Ruminant Infectious Disease Prevention and Control (East), Ministry of Agriculture and Rural Affairs, College of Animal Science and Veterinary Medicine, Shenyang Agricultural University, Shenyang 110866, China.; ^2^ Research Unit for Pathogenic Mechanisms of Zoonotic Parasites, Chinese Academy of Medical Sciences, Shenyang 110866, China.

## Abstract

Neutrophils are essential in combating invading pathogens such as *Plasmodium* parasites, but the participation of their subpopulations and mechanisms in resistance to parasite infection are not fully understood. Our study identified a marked increase in Ly6G^+^ neutrophils in response to *P. berghei* ANKA infection. Depletion of these cells rendered mice more susceptible to infection. Elevated interleukin-17 (IL-17) levels, which increased the Ly6G^+^ neutrophil population, were also found to contribute to this protective effect. IL-17 depletion led to reduced neutrophil numbers and increased susceptibility. Furthermore, dihydroartemisinin (DHA) treatment enhanced neutrophil-mediated immune responses through up-regulation of CD18 and CXCR4 factors. These findings revealed key mechanisms of neutrophil and IL-17 interactions in malaria protection and highlighted DHA’s potential to promote neutrophil function in combating malaria.

## Introduction

Neutrophils, the most abundant leukocytes in humans, are crucial in the immune response to malaria caused by *Plasmodium* parasites. They are among the first responders at the infection site, using various mechanisms to combat the parasites. Neutrophils, the most prevalent leukocytes in human circulation, play a pivotal role in the immune response to malaria, a disease caused by *Plasmodium* parasites [1,2]. A principal mechanism involves phagocytosis, facilitated by the release of neutrophil extracellular traps (NETs) [3,4], through which neutrophils engulf and destroy *Plasmodium*-infected red blood cells (iRBCs), thereby diminishing the parasitemia. Additionally, neutrophils possess the capability to eliminate *Plasmodium* by producing reactive oxygen species (ROS). Upon activation, neutrophils generate ROS, which are deleterious to the parasites. As commonly observed, patients with severe malaria or cerebral malaria exhibit elevated levels of ROS, which may be related to the severity of the disease [5,6]. Further, neutrophils can recruit immune cells, including macrophages, monocytes, and lymphocytes, by secreting a variety of cytokines [7].

Dihydroartemisinin (DHA), a primary anti-malarial agent, exhibits notable immunomodulatory properties [8–10]. The anti-inflammatory property of DHA can be observed in cases of acute lung injury caused by lipopolysaccharide by reducing levels of tumor necrosis factor-α (TNF-α), which is a pro-inflammatory cytokine [11]. Additionally, it modulates immune cell activity by enhancing the function of T regulatory cells, thereby contributing to the maintenance of immune homeostasis [12]. However, the precise underlying mechanisms of these effects remain to be elucidated.

The expression levels of CD18 and CXCR4 are critical in modulating neutrophil functionality. CD18, a key subunit of the integrin family, plays a predominant role in the migration of neutrophils from the peripheral blood to other tissues, thereby facilitating an effective response to infection [13–16]. Our previous studies indicate that DHA treatment significantly increases the proportion of CD18^+^ Ly6G^+^ neutrophils [17]. CXCR4, a chemokine receptor, is essential for regulating neutrophil trafficking through its interaction with the ligand CXCL12 (SDF-1). In malaria, previous study indicated that CXCR4 acts as a crucial host factor facilitating the transformation of *Plasmodium* sporozoites into exoerythrocytic form in the liver, thereby presenting a potential target for malaria prophylaxis [18]. Additionally, inhibition of CXCR4 in *Plasmodium chabaudi* CR-infected mice has been shown to elevate parasitemia [19].

In this study, we found that Ly6G^+^ neutrophils are crucial in resisting *Plasmodium berghei* ANKA infection, and interleukin-17 (IL-17) can selectively promote the expansion of Ly6G^+^ subpopulation. Depletion of either Ly6G^+^ neutrophils or IL-17 results in higher susceptibility to parasite infection. We also discovered that DHA enhances neutrophil-mediated immunity through CD18 up-regulation. These findings reveal the crosstalk between Ly6G^+^ neutrophils and IL-17, and further highlight DHA’s role in boosting immune responses.

## Results

### The essential role of Ly6G^+^ neutrophils in resistance to *P. berghei* ANKA infection

**Fig. 1.**
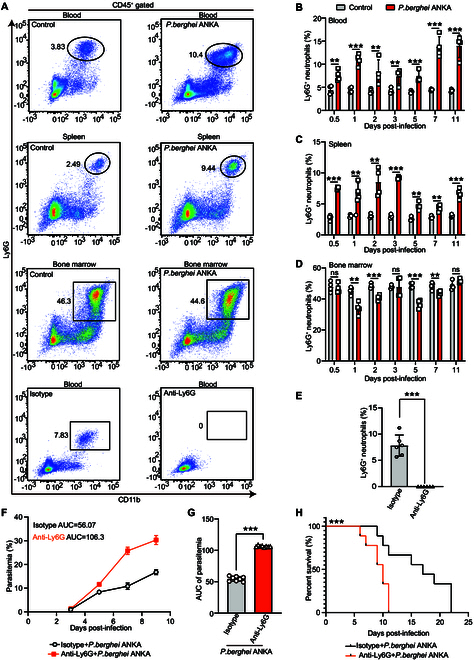
Ly6G^+^ neutrophils were elevated and conferred resistance to infection with *P. berghei* ANKA. (A) Representative scatterplots of positive cells in the peripheral blood, spleen, and bone marrow of mice after cell surface CD45^+^ CD11b^+^ Ly6G^+^ staining on different days in the control groups, *P. berghei* ANKA groups, isotype groups, and anti-Ly6G mAb groups. (B) The Ly6G^+^ neutrophils in the peripheral blood increased significantly after infection (*n* = 4). (C) The Ly6G^+^ neutrophils in the spleen increased significantly in infected mice (*n* = 4). (D) No significant change of the Ly6G^+^ neutrophils in the bone marrow after infection (*n* = 4). (E) The anti-Ly6G mAb completely depleted Ly6G^+^ neutrophiles from the circulation compared to the isotype antibody (*n* = 6). (F) The parasitemia of infected mice after injection of anti-Ly6G mAb and isotype mAb was measured every other day. (G) The parasitemia was indicated by AUC with mean ± SD. Compared to the isotype mAb-treated group, the parasitemia of mice injected with anti-Ly6G mAb was much higher (*n* = 10, *P* < 0.0001). (H) Pretreatment with the anti-Ly6G mAb shortened the survival time of infected mice (*n* = 10, *P* < 0.0001). ns: *P* > 0.05; *P < 0.05; **P < 0.01; ***P < 0.001.

To investigate the immune response of Ly6G^+^ neutrophils to malaria, we utilized *P. berghei* ANKA to infect BALB/c mice, with uninfected BALB/c mice serving as controls. In comparison with uninfected controls, *P. berghei* ANKA-infected mice showed significantly higher levels of Ly6G^+^ neutrophils in peripheral blood and spleens at various time points (Fig. 1A to C). Specifically, on the first day after infection, the infected group displayed 10.4% Ly6G^+^ neutrophils compared to 3.83% in the control group, illustrating a robust increase in Ly6G^+^ neutrophils early in the infection (Fig. 1B). Notably, this elevation in Ly6G^+^ neutrophils did not occur in the bone marrow, indicating that the increase was specific to the peripheral blood and spleen (Fig. 1A and D). To investigate the role of Ly6G^+^ neutrophils in *P. berghei* ANKA-infected mice, we used a specific antibody to completely deplete Ly6G^+^ neutrophils from the circulation of the *P. berghei* ANKA-infected mice (Fig. 1A and E). Subsequently, we found that parasitemia in the anti-Ly6G-treated group was significantly higher compared to the isotype control group (Fig. 1F). The area under the curve (AUC) is 106.3 for the anti-Ly6G group and 56.07 for the isotype control group, indicating a greater parasite burden in the anti-Ly6G-treated mice (Fig. 1G). Furthermore, the depletion of Ly6G^+^ neutrophils in the infected mice resulted in premature mortality (Fig. 1H). These findings underscore the critical role of Ly6G^+^ neutrophils in combating *P. berghei* ANKA infection.

### IL-17 is essential in resistance to *P. berghei* ANKA infection

**Fig. 2.**
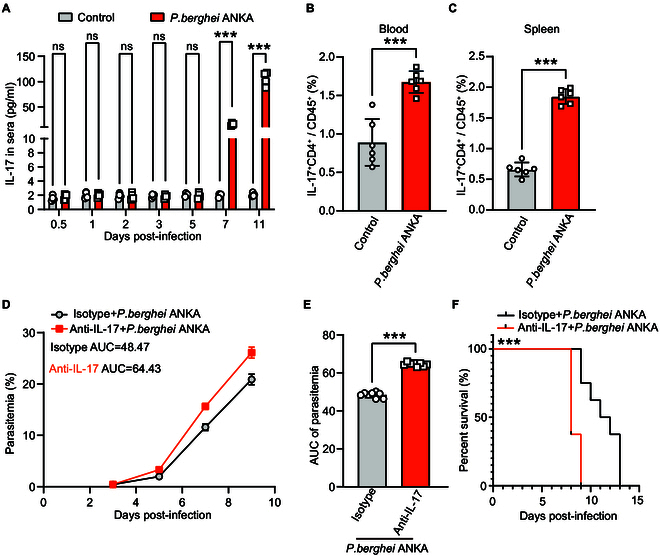
IL-17^+^ CD4^+^ T cells are closely associated with immune resistance to *P. berghei* ANKA infection. (A) The levels of IL-17 in the sera of infected increased significantly on days 7 and 11 (*n* = 4). (B) The IL-17^+^ CD4^+^ T cells in the peripheral blood increased significantly on days 7 and 11 after infection (*n* = 6, *P* = 0.0002). (C) The IL-17^+^ CD4^+^ T cells in the spleen increased significantly on days 7 and 11 after infection (*n* = 6, *P* < 0.0001). (D) The parasitemia of infected mice after injection of an anti-IL-17 mAb and an isotype mAb was measured every other day. (E) The parasitemia was indicated by AUC with mean ± SD. Compared with the isotype mAb group, the parasitemia of mice injected with the anti-IL-17 mAb was much higher (*n* = 10, *P* < 0.0001). (F) Pretreatment with anti-IL-17 mAb significantly shortened the survival of the *P. berghei* ANKA-infected mice (*n* = 10, *P* < 0.0001). ns: *P* > 0.05; *P < 0.05; **P < 0.01; ***P < 0.001.

To investigate the cause for the elevation of Ly6G^+^ neutrophils after *P. berghei* ANKA infection, we assessed the levels of 10 cytokines in the sera of infected mice compared to uninfected mice (Fig. S1). The levels of IL-17 in the control and infected groups show no significant difference on days 1, 2, 3, and 5 after infections. However, the IL-17 levels in the infected mice were significantly higher compared to that of the control group by days 7 and 11 after infection (*P* < 0.0001) (Fig. 2A). Correspondingly, we found a significant increase in the percentage of IL-17^+^ CD4^+^ cells in *P. berghei* ANKA-infected mice compared to the control mice in both peripheral blood and spleen (*P* = 0.0002 and *P* < 0.0001) (Fig. 2B and C). Further, the anti-IL-17-treated group showed higher parasitemia levels over the course of the infection compared to the isotype control group (Fig. 2D). Equivalently, the AUC for parasitemia is significantly greater in the anti-IL-17 group compared to the isotype control group (Fig. 2E). The survival rate of mice in the anti-IL-17 group is significantly lower compared to the isotype control group (*P* < 0.0001) (Fig. 2F), indicating that IL-17 plays a key role in immunity to the infection of *P. berghei* ANKA.

### IL-17 engages in crosstalk with Ly6G^+^ neutrophils

**Fig. 3.**
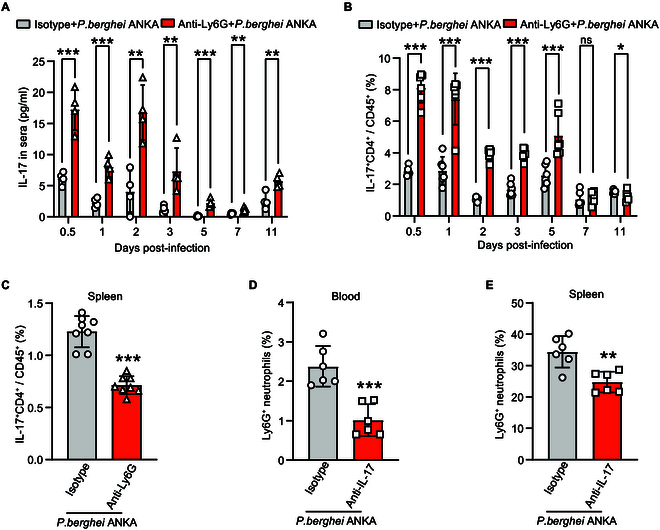
Ly6G^+^ neutrophils crosstalk with IL-17^+^ T cells. (A) The mice were injected with anti-Ly6G mAb subsequently infected with *P. berghei* ANKA, and the levels of IL-17 in the sera increased significantly on the early days after infection (*n* = 4). (B) The mice were injected with anti-Ly6G mAb subsequently infected with *P. berghei* ANKA, and the levels of IL-17^+^ CD4^+^ T cells in the blood increased significantly on the early days after infection but showed no difference on days 7 and 11 (*n* = 6). (C) The number of IL-17^+^ CD4^+^ T cells in the spleen of infected mice decreased significantly after treatment with an anti-Ly6G mAb (*n* = 6, *P* < 0.0001). (D and E) Anti-IL-17 mAb reduced the number of Ly6G^+^ neutrophils in the peripheral blood and spleen of *P. berghei* ANKA-infected mice (*n* = 6, *P* = 0.0005 and *P* = 0.0028). ns: *P* > 0.05; *P < 0.05; **P < 0.01; ***P < 0.001.

To further analyze whether Ly6G^+^ neutrophils regulate IL-17 responses in *P. berghei* ANKA-infected mice, we assessed IL-17 levels after the depletion of Ly6G^+^ neutrophils. The levels of IL-17 in the control and anti-Ly6G-treated groups showed significant differences on various days after infection. It was significantly elevated in the anti-Ly6G-treated group compared to the control group on days 1, 2, 3, 5, and 11 after infection (*P* values: 0.0007, 0.0028, 0.0073, 0.0007, and 0.0055, respectively) (Fig. 3A). The depletion of Ly6G^+^ neutrophils caused a shift in IL-17 levels in *P. berghei* ANKA-infected mice, with elevated levels observed early in the infection instead of the late stage (Fig. 3A). Consistent with previous findings, we observed a significant increase in the levels of IL-17^+^ CD4^+^ T cells from 0.5 to 5 days after infection in the infected mice following the depletion of Ly6G^+^ neutrophils (Fig. 3B). By days 7 and 11, the levels of these cells showed no significant difference compared to controls (Fig. 3B). However, we found a significant decrease in the percentage of IL-17^+^ CD4^+^ T cells in the spleen of anti-Ly6G-treated mice compared to control mice (*P* < 0.0001) (Fig. 3C). We next demonstrated the significant impact of IL-17 on the Ly6G^+^ neutrophils in the infected mice. Flow cytometry analysis revealed that the percentage of Ly6G^+^ neutrophil cells in the peripheral blood was reduced in the anti-IL-17-treated group compared to the isotype control group (*P* = 0.0005) (Fig. 3D). The anti-IL-17-treated group exhibited a significantly lower percentage of splenic Ly6G^+^ neutrophil cells (*P* = 0.0028) (Fig. 3E) compared to the isotype control group. These results suggest that IL-17 engaged in crosstalk with Ly6G^+^ neutrophils in *P. berghei* ANKA infection.

### DHA enhanced the crosstalk between Ly6G^+^ neutrophils and other immune cells

**Fig. 4.**
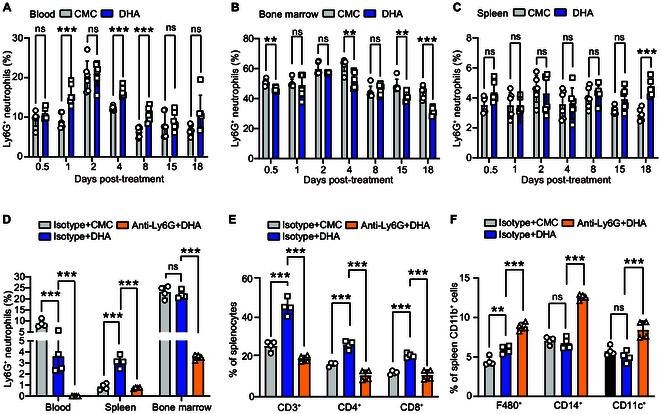
DHA regulates the host immune response through Ly6G^+^ neutrophils. (A) A significant increase in Ly6G^+^ neutrophils was observed in the peripheral blood of mice on days 1, 4, and 8 after treatment with DHA (*n* = 6, *P* = 0.0007, *P* = 0.0001, and *P* = 0.0008). (B) Ly6G^+^ neutrophils in the bone marrow decreased significantly after DHA treatment on days 1 (*P* = 0.0029), 4 (*P* = 0.0030), 15 (*P* = 0.0029), and 18 (*P* = 0.0002) (*n* = 6). (C) Ly6G^+^ neutrophils in the spleen increased significantly after DHA treatment on day 18 (*n* = 6, *P* = 0.0004). (D) Ly6G^+^ neutrophils in the peripheral blood, bone marrow, and spleen of the mice injected with anti-Ly6G mAb and DHA decreased significantly compared to those treated with an isotype mAb and DHA (*n* = 4, *P* < 0.0001). (E) CD3^+^, CD4^+^, and CD8^+^ T cells in the spleen decreased significantly in the anti-Ly6G mAb group compared with the isotype group (*n* = 4, *P* < 0.0001). (F) F4/80^+^ CD11b^+^, CD14^+^CD11b^+^, and CD11c^+^ CD11b^+^ cells in the spleen increased significantly in the anti-Ly6G mAb group compared with the isotype group (*n* = 4, *P* < 0.0001, *P* < 0.0001, and *P* = 0.0005). ns: *P* > 0.05; *P < 0.05; **P < 0.01; ***P < 0.001.

We previously observed that DHA can induce Ly6G^+^ neutrophil expansion in mice [17]. In this study, we also found an early increase of Ly6G^+^ neutrophils in the peripheral blood after DHA treatment (Fig. 4A), accompanied by a reduction in neutrophils in the bone marrow (Fig. 4B). The results suggest that DHA might promote neutrophil migration from the bone marrow to the peripheral blood. Moreover, we observed that with prolonged DHA gavage, the proportion of splenic Ly6G^+^ neutrophils gradually increased compared to the control group (Fig. 4C). These results indicated that DHA has a regulatory effect on neutrophils. To investigate whether Ly6G^+^ neutrophils play a decisive role in DHA-induced immunomodulation, we depleted neutrophils in DHA-gavaged mice and then analyzed the changes in the proportions of other immune cells (Fig. 4D). Depletion of Ly6G^+^ neutrophils led to a significant reduction in the proportions of CD3^+^, CD4^+^, and CD8^+^ T cells in the spleen compared to the isotype + DHA and isotype + carboxymethyl cellulose (CMC) groups (*P* < 0.0001) (Fig. 4E). However, depletion of Ly6G^+^ neutrophils resulted in a significant increase in the proportions of macrophages, monocytes, and dendritic cells in the spleen (Fig. 4F). To confirm that DHA can directly promote the differentiation of T cells into T helper 17 (T_H_17) cells, we sorted CD4^+^ T cells and polarized them into T_H_17 cell subsets following in vitro culture. After stimulating these cells with DHA and *P. berghei* ANKA, we observed a significant increase in the proportion of T_H_17 cells in the group prestimulated with DHA before exposure to *P. berghei* ANKA (*P* < 0.0001) (Fig. S2). These findings suggest that both DHA and *P. berghei* ANKA can directly target CD4^+^ T cells, leading to an elevated production of IL-17.

### DHA activated Ly6G^+^ neutrophils by inducing the expression of CD18 and CXCR4

**Fig. 5.**
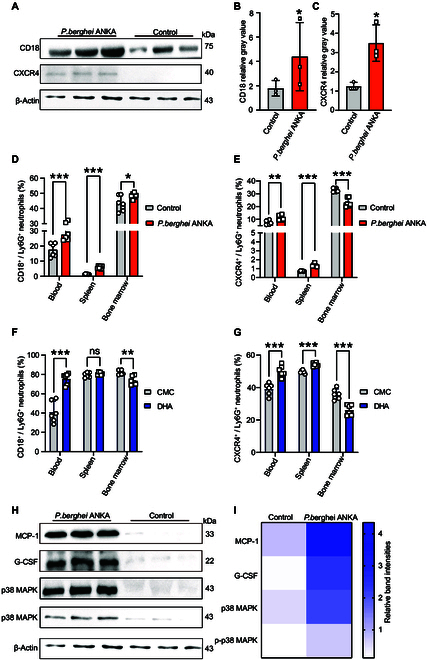
Both DHA treatment and *P. berghei* ANKA infection significantly elevated the populations of CD18^+^ and CXCR4^+^ Ly6G^+^ neutrophils in blood and spleen tissues. (A) The expression of CD18 and CXCR4 was significantly increased in *P. berghei* ANKA infection. (B and C) The bands (A) were quantified using the ImageJ software, and β-actin was employed as the internal control. The results are presented as the mean ± SEM (*n* = 3). (D and E) The proportion of CD18^+^ Ly6G^+^ neutrophils and CXCR4^+^ Ly6G^+^ neutrophils increased in the peripheral blood and spleen of infected mice. However, with a decrease in the proportion of CXCR4^+^ Ly6G^+^ neutrophils in the bone marrow, the proportion of CD18^+^ Ly6G^+^ neutrophils increased. (F and G) The proportion of CD18^+^ Ly6G^+^ neutrophils and CXCR4^+^ Ly6G^+^ neutrophils were markedly increased in the peripheral blood and spleen but decreased in the bone marrow in the DHA-treated mice (*n* = 6). (H) The levels of MCP-1, G-CSF, and p38 MAPK proteins and the phosphorylation level of p38 MAPK protein were detected via Western blotting with protein-specific antibodies. (I) The bands (H) were quantified using the ImageJ software, and β-actin was employed as the internal control. The results are presented as the mean ± SEM (*n* = 3). ns: *P* > 0.05; *P < 0.05; **P < 0.01; ***P < 0.001.

Our previous study found that splenic Ly6G^+^ neutrophils expressed CD18 and CXCR4 following DHA treatment by using single-cell RNA sequencing [17]. To further investigate the roles of CD18 and CXCR4 in neutrophils, we isolated neutrophils from *P. berghei* ANKA-infected mice as well as from uninfected controls. The expression of CD18 and CXCR4 was assessed using protein-specific antibodies. Both CD18 (Fig. 5A and B) and CXCR4 (Fig. 5A and C) were found highly expressed on neutrophils of *P. berghei* ANKA-infected mice, with CXCR4 expression being exclusive to the neutrophils of infected mice. We further investigated the proportion of CD18- and CXCR4-expressing neutrophils during *P. berghei* ANKA infection. Compared to the control mice, the *P. berghei* ANKA-infected mice had significantly higher proportion of CD18^+^ Ly6G^+^ neutrophils in the blood and spleen (Fig. 5D), as well as a significant increase in the bone marrow (*P* = 0.0173). Similarly, the percentage of CXCR4^+^ Ly6G^+^ neutrophils was also significantly higher in *P. berghei* ANKA-infected mice compared to the controls in both the blood and spleen (Fig. 5E).

**Fig. 6.**
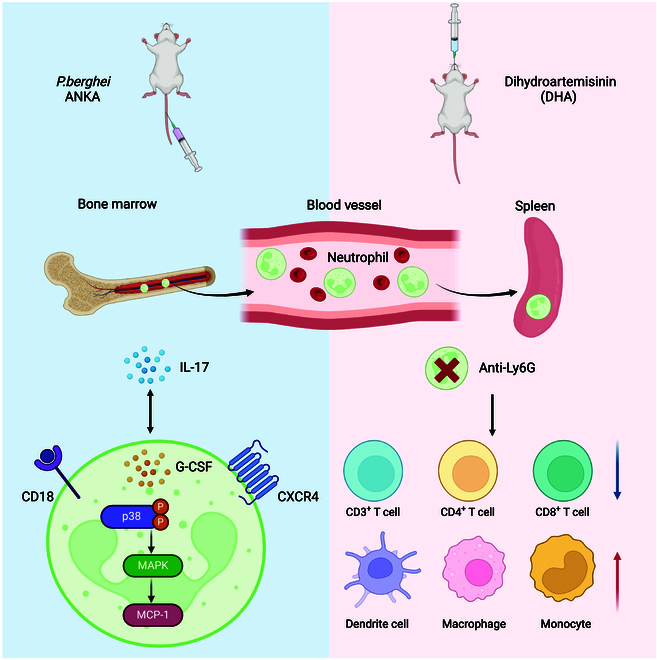
Both Ly6G^+^ neutrophils and IL-17^+^ T cells are key cell subsets in resistance to *Plasmodium* infection. Ly6G^+^ neutrophils and IL-17 have protective effects during *P. berghei* ANKA infection, and they can regulate each other. DHA promotes migration of Ly6G^+^ neutrophils from the bone marrow to peripheral blood and spleen by up-regulating the expression levels of CD18 and CXCR4, which is regulated through the p38 MAPK/MCP-1 signal axis.

To investigate whether DHA has a similar promotive effect on the aforementioned phenotypes, we assessed the proportion of CD18- and CXCR4-expressing neutrophils in uninfected mice gavaged with DHA compared to those receiving CMC as a control. DHA-treated mice had significantly more CD18^+^ Ly6G^+^ neutrophils in the blood than control mice (Fig. 5F). In contrast, mice treated with DHA had lower levels of CD18^+^ Ly6G^+^ neutrophils in their bone marrows (Fig. 5F). These results suggest that DHA promotes the migration of CD18^+^ Ly6G^+^ neutrophils from the bone marrow to the peripheral blood. Similarly, DHA-treated mice had significantly more CXCR4^+^ Ly6G^+^ neutrophils in their blood and spleens than CMC-treated mice (Fig. 5G), whereas that in the bone marrow showed lower percentages (*P* = 0.0004). To elucidate the molecular mechanisms underlying the up-regulation of CD18 and CXCR4, we employed various specific antibodies to screen for alterations in key molecular signaling pathway in neutrophils before and after infection. We observed a significant up-regulation of expression of granulocyte activation-associated cytokines, monocyte chemoattractant protein-1 (MCP-1) (Fig. 5H and I) and granulocyte colony-stimulating factor (G-CSF) (Fig. 5H and I), in neutrophils after infection. This up-regulation is likely due to the activation of the mitogen-activated protein kinase (MAPK)/p38 phosphorylation signaling pathway in neutrophils (Fig. 5H and I). Collectively, these results demonstrate that both DHA treatment and *P. berghei* ANKA infection significantly alter the populations of CD18^+^ Ly6G^+^ neutrophils and CXCR4^+^ Ly6G^+^ neutrophils in the blood and spleen (Fig. 6).

## Discussion

In this study, our findings indicate a significant increase in neutrophils, characterized by the markers CD45^+^ CD11b^+^ Ly6G^+^, within the peripheral blood and spleen of mice infected with *P. berghei* ANKA at multiple time points following infection. This observation aligns with other studies indicating that neutrophils rise following *Plasmodium* infection. For example, a transcriptome and metabolome analysis with samples from patients of *Plasmodium vivax* infection showed a significant increase of neutrophils in peripheral blood [20]. Our findings suggest that Ly6G^+^ neutrophils play a protective role during *P. berghei* ANKA infection, as depletion of Ly6G^+^ neutrophils using an anti-Ly6G monoclonal antibody (mAb) resulted in significantly increased parasitemia and shortened survival times. These results are consistent with the observation that neutrophils can phagocytize *Plasmodium falciparum* merozoites [21], further supporting the protective function of neutrophils during malaria.

We observed a significant up-regulation of Ly6G^+^ neutrophils following infection, which prompted us to investigate the mechanisms underlying this phenomenon. A significant increase in IL-17 and MCP-1 levels was also detected in the sera of infected mice. However, only IL-17 was selected for further study due to its critical role as a cytokine in neutrophil recruitment and activation [22–24]. In contrast, MCP-1 secreted by neutrophils primarily functions to recruit monocytes to specific sites [25], rather than serving as a key driver in neutrophil proliferation or accumulation. The role of IL-17 in *P. berghei* ANKA infection was confirmed by using anti-IL-17 mAb, which resulted in increased parasitemia and reduced survival. These results, along with the observed mutual regulation between Ly6G^+^ neutrophils and IL-17, highlight the interdependent relationship between these factors in the context of malaria infection.

DHA, a derivative of artemisinin, is well known for its antimalarial efficacy [26]. Our previous work has demonstrated that DHA significantly increases neutrophil numbers in the spleen [12,17]; however, the specific subpopulations and underlying mechanisms remain unclear. In this study, based on previous single-cell sequencing results [17], we identified Ly6G^+^ neutrophils as the specific cell subpopulation induced by DHA and analyzed the regulatory effects of DHA on this subset through a time-series approach. We observed that the most pronounced changes occurred on day 18 following DHA administration. Consequently, we further assessed the immunomodulatory activity of DHA on other immune cells at this time point after Ly6G^+^ neutrophil depletion. This resulted in significant reductions in CD3^+^, CD4^+^, and CD8^+^ lymphocytes in the spleen, alongside with the increase of myeloid cell subsets. These findings suggest that Ly6G^+^ neutrophils play a significant role in the immunomodulatory effects induced by DHA. However, this study was conducted in the mice infected with *P. berghei* ANKA; thus, we have not explored these effects on exposed populations. This needs to be studied in further detail in the future.

To elucidate the molecular mechanisms underlying these effects, we examined the expression of CD18 and CXCR4 marker proteins, which are associated with neutrophil function. CD18 integrins, present in all leukocytes, are crucial for immune responses, including neutrophil recruitment [27]. We observed increased CD18^+^ CD11b^+^ Ly6G^+^ cells in the blood and spleen following DHA treatment and *P. berghei* ANKA infection, with decreased levels in the bone marrow. This suggests that CD18 regulates neutrophil dynamics in response to DHA treatment and parasite infection. IL-17 promotes neutrophil recruitment by up-regulating CD18 expression [28]. It can also up-regulate ICAM-1 expression, providing additional binding sites for CD18 on neutrophils, thereby enhancing neutrophil adhesion to vascular endothelium and facilitating their migration to inflammatory sites [29]. CXCR4 is involved in neutrophil migration, with CXCL12 as the major ligand [30]. The knockout of CXCR4 in mice leads to an increased population of neutrophils in the bloodstream, originating from the bone marrow [31]. IL-17 can up-regulate the expression of CXCR4, promoting neutrophil migration to inflammatory sites [32]. Our findings indicate that both DHA treatment and *P. berghei* ANKA infection affect CXCR4 expression, influencing neutrophil distribution and function.

p38 MAPK belongs to the MAPK superfamily and is involved in many important physiological processes. The pathway is activated in response to external stimuli, such as inflammation, and is involved in the development of various inflammatory diseases as well as the regulation of cytokine release [33,34]. Macrophage chemoattractant protein-1 (MCP-1) is one of the key molecules downstream of the p38 MAPK-activated signaling pathway whose main function is to recruit inflammatory cells to damaged organs and tissues [35]. G-CSF can promote the expression of CD18 on granulocytes through the p38 MAPK-mediated signaling pathway [36]. Another study also demonstrated that p38 MAPK activation was essential for CD18 expression [37]. In line with previously reported data, the MAPK and nuclear factor κB (NF-κB) signaling pathways were suppressed in CXCR4^−/−^ mice [38]. Previous studies have reported that MCP-1 (it can interact with CD18), G-CSF, and p38 MAPK phosphorylation play critical roles in regulating the expression of CD18 and CXCR4 [39]. IL-17 can also promote neutrophil accumulation in the lungs through the p38 MAPK signaling pathway [40]. Therefore, we extracted the protein of Ly6G^+^ neutrophils after infection with *P. berghei* ANKA and found that the expression of p38 MAPK, p-p38 MAPK, G-CSF, and MCP-1 was significantly increased compared to the uninfected group. This suggests that IL-17 may stimulate the expression of CD18 and CXCR4 through p38 MAPK, thereby leading to an increase in Ly6G^+^ neutrophils.

In summary, our study highlights the protective role of neutrophils during *P. berghei* ANKA infection and the significant impact of IL-17 and DHA on neutrophil regulation. The mutual regulation between Ly6G^+^ neutrophils and IL-17, along with the involvement of CD18 and CXCR4, provides insights into the complex interactions governing immune responses in malaria. These findings contribute to a better understanding of the mechanisms by which neutrophils, cytokines, and antimalarial drugs like DHA interact to influence infection outcomes.

## Materials and Methods

### Animals

Female BALB/c mice, aged 6 to 8 weeks, were obtained from Liaoning Changsheng Biological Technology Company and adapted to reversed light–dark cycles for 1 week before the experiment. Procedures followed Shenyang Agricultural University’s guidelines and were approved by its Ethical Committee (permit no. SYXK<Liao>2021-0010).

### Drugs and antibodies

To make 0.5% CMC, 0.25 g of CMC was dissolved in 50 ml of preheated distilled water. For 0.1 mg/ml DHA preparation, 0.3 g of DHA was added in 30 ml of 0.5% CMC and stirred at 4 °C in the dark until fully dissolved [41]. Each mouse was administered a gavage dose of 2 mg of DHA. The anti-mouse Ly6G antibody (catalog no. L280, Leinco, USA) was diluted to a concentration of 2 mg/ml using normal saline. Similarly, the isotype control antibody (rat IgG2a, catalog no. I-1177, Leinco) was also diluted to 2 mg/ml with normal saline. The anti-mouse IL-17 antibody (catalog no. BP0173, BioXcell, USA) was diluted to 2 mg/ml with normal saline. Similarly, the isotype control antibody (mouse IgG1, catalog no. BE0083, BioXcell) was also diluted to 2 mg/ml with normal saline. All antibodies are described in Table S1.

### In vivo Ly6G and IL-17 depletion

To deplete Ly6G and IL-17 in vivo, BALB/c mice were administered with anti-Ly6G mAb (200 μg) and IL-17 mAb (200 μg) via tail vein injection. Control group BALB/c mice received intraperitoneal injections of the corresponding isotype control immunoglobulin G (IgG). The efficacy of depletion of Ly6G^+^ and IL-17^+^ cells in the treated mice was verified through flow cytometry analysis. Subsequently, mice were intraperitoneally injected with 1 × 10^5^ *P. berghei* ANKA parasites. Parasitemia was monitored every other day using Giemsa-stained blood smear. Mice that received anti-Ly6G mAb were again assessed by flow cytometry to confirm the absence of Ly6G^+^ cells. Following confirmation, these mice were orally administered DHA or CMC without infection. Upon successful depletion of Ly6G, flow cytometry was employed to detect changes in the proportions of lymphocytes and myeloid cell subsets and peripheral blood of the mice.

### Flow cytometry—sample preparation

Two groups of BALB/c mice were randomly assigned to receive either CMC (200 μl) or DHA (2 mg) for 1, 2, 4, 8, 15, or 18 days without infection. After preparing single-cell suspensions from different tissues, erythrocytes were lysed using erythrocyte lysate after filtration through a 70-μm filter. Anti-mouse CD16/32 antibody (1:50, catalog no. 101320, BioLegend, USA) was added to cells to block nonspecific binding by incubating in the dark for 15 min. The antibody solution, prepared as described below, was then added for 30 to 35 min at 4 °C without light and washed with phosphate-buffered saline (PBS) 3 times [41]. 7-AAD (7-aminoactinomycin D,1:300, catalog no. 420404, BioLegend) was used for 3 to 5 min.

All immune cells were evaluated by staining with anti-CD45-phycoerythrin (PE)/Cyanine7 (1:20, catalog no. E-AB-F1136H, Elabscience, China). Neutrophils were evaluated by staining with anti-CD11b-allophycocyanin (APC) (1:20, catalog no. E-AB-F1081E, Elabscience) and anti-Ly6G-PE (1:20, catalog no. E-AB-F1108D, Elabscience). CD18^+^ and CXCR4^+^ cells were evaluated by staining with anti-CD18-PE (1:20, catalog no. 101407, BioLegend, USA), anti-CXCR4-Brilliant Violet 605 (1:80, catalog no. 146519, BioLegend), anti-CD11b-APC (1:20, catalog no. E-AB-F1081E, Elabscience), and anti-Ly6G-fluorescein isothiocyanate (FITC) (1:20, catalog no. E-AB-F1108C, Elabscience). All lymphocyte subsets were evaluated by staining with anti-CD3-FITC (1:20, catalog no. E-AB-F1013C, Elabscience), anti-CD4-PE (1:20, catalog no. E-AB-F1353D, Elabscience), and anti-CD8-APC (1:20, catalog no. E-AB-F1104E, Elabscience). All myeloid cell subsets were evaluated by staining with ant-F4/80-FITC (1:20, catalog no. E-AB-F0995C, Elabscience), anti-CD14-PE (1:20, catalog no. E-AB-F1176D, Elabscience), and anti-CD11c-Elab Fluor Violet 450 (1:20, catalog no. E-AB-F0991Q, Elabscience).

### Intracellular staining—flow cytometry

The single cells were cultured in 12-well plates containing 1640 medium (catalog no. R8758, Sigma, USA) without lysis of the erythrocytes. The cell number was adjusted to 1 × 10^8^ per ml. After adding 2 μl of the cell stimulator (catalog no. E-CK-A091, Elabscience) to the cells for 2 h, 1 μl of the protein transport inhibitor (catalog no. E-CK-A091, Elabscience) was then added to each culture plate. After the cells were cultured for 5 h, they were collected into centrifuge tubes for erythrolysis. All splenic cells were stained with anti-CD4-FITC (1:20, catalog no. E-AB-F1353C, Elabscience) at 4 °C for 30 min. Then, 200 μl of True-Nuclear Fixation Buffer (catalog no. 424401, BioLegend) was added to the cells, washed 3 times with True-Nuclear Perm Buffer (catalog no. 424401, BioLegend), and stained with secondary antibody anti-IL-17-PE (1:20, catalog no. E-AB-F1199D, Elabscience).

### Magnetic bead separation of neutrophils

Splenic cells were prepared without RBC Lysis buffer. After counting with a hemocytometer, MojoSort Buffer was used to adjust the cells to 1 × 10^8^ cells/ml. The Biotin-Antibody Cocktail (catalog no. 480058, BioLegend) was added at a 1:10 ratio and mixed on a vortex shaker for 12 min. MojoSort Buffer was used to wash cells and then centrifuged at 4 °C for 6 min at 280*g*. Streptavidin nanobeads (catalog no. 480058, BioLegend) were added at a 1:10 ratio and mixed. Subsequently, 1 ml of MojoSort Buffer was add and centrifuged at 280*g* for 5 to 8 min. Last, the cell suspension was incubated on a magnetic bar on ice for 3 to 5 min, retaining the nonadsorbed portion. The neutrophils formed the precipitate after centrifugation. The sorted neutrophils were stained with anti-Ly6G-PE and anti-CD11b-APC antibodies and then sorting purity was assessed using flow cytometry.

### Western blotting

Mouse neutrophil proteins were extracted using Cell Total Protein Lysis Buffer [catalog no. C500001, Sangon Biotech (Shanghai) Co. Ltd., China] following the kit’s instructions. The proteins were then transferred to a polyvinylidene difluoride (PVDF) membrane using the wet transfer method. Then, the membrane was incubated with 5% skimmed milk solution in phosphate-buffered saline with 0.05% Tween 20 (PBST) at 37 °C for 1 h. Antibodies were then incubated with the samples overnight at 4 °C (antibody details are provided in Table S1). Subsequently, the membrane was washed with PBST for 30 min and incubated with corresponding species-specific secondary antibodies conjugated to horseradish peroxidase (HRP) (1:1000, catalog nos. A0208 and A0216, Beyotime), diluted in PBST, for 45 min at 37 °C. After 30 min of washing with PBST, the enhanced chemiluminescence reagent was prepared, and proteins were detected on the PVDF membrane. Results were quantified using ImageJ software.

### Statistical analysis

Data comparisons between the 2 groups were conducted using the Student’s *t* test to identify significant differences, while analysis of variance (ANOVA) was used for 3 or more groups. Results are shown as means, with significance indicated numerically, and analyzed with GraphPad Prism. Uncropped immunoblot images were listed in Fig. S3, flow cytometry gate was detailed in Fig. S4, and the details of antibodies were listed in Table S1.
