# Supplementary material for: Ly6G+ Neutrophils and Interleukin-17 Are Essential in Protection against Rodent Malaria Caused by Plasmodium berghei ANKA
Source: Research (Wash D C). 2024 Dec 19;7:0559. doi: 10.34133/research.0559 (PMC11658117; doi:10.34133/research.0559)
Supplement: Supplementary 1 — Methods Figs. S1 to S4 Table S1 [file research.0559.f1.docx]

**Supplementary materials**

**Supplementary Methods**

**Cytometric bead array (CBA)**

The concentrations of cytokines, specifically IL-17, in the sera of infected BALB/c mice were quantified using a CBA mouse inflammation kit (Cat. No. BS-MC01, Beadstar Biotechnology, China). Sera samples were collected on days 1, 2, 3, 5, 7 and 11 post-infections through centrifugation at 1, 000×g for 20 min. Subsequently, 50 μL of captured beads was combined with 50 μL of serum sample and 50 μL of mouse inflammation PE detection reagent, followed by incubation at 22 ± 2 ℃ in the dark for 2 h. Following washing with 1 mL of washing buffer, the samples were centrifuged at 300×g for 5 min. The pellets were resuspended in 300 μL of washing buffer and analyzed using a FACS Aria III with FACS Diva software (BD Biosciences).

**Th17 cell differentiation experiments**

On day 0, 12-well plates were coated with Anti-mouse CD3 (Cat. No. 100339, Biolegend) at a concentration of 5 µg/mL and incubated overnight at 4 °C. The wells were then gently emptied and washed three times with sterile PBS. Sorted naive CD4^+^ T cells were seeded at a density of 2×10^5^ cells/mL into the coated wells. The following reagents were added to the culture: 5 µg/mL Anti-mouse CD28 (Cat. No. 102116, Biolegend), 50 ng/mL Recombinant mouse IL-6 (Cat. No. P00158, Solarbio), 1 ng/mL Recombinant human TGF-β1 (Cat. No. P00199, Solarbio), 5 ng/mL Recombinant mouse IL-23 (Cat. No. P00230, Solarbio), 10 µg/mL Anti-mouse IL-4 (Cat. No. 504122, Biolegend), and 10 µg/mL Anti-mouse IFN-γ (Cat. No. 505834, Biolegend). The cells were cultured for 4 days. On day 3, 5 mL of fresh medium containing the same concentrations of antibodies and cytokines as on day 0 was carefully added to each well. On day 4, the cells were washed once, and 500 ng/mL PMA (Cat. No. 423303, Biolegend) and 500 ng/mL ionomycin (Cat. No. 423303, Biolegend) were added to the complete medium for stimulation, along with Brefeldin A (Cat. No. 420601, Biolegend), DHA or *P. berghei* ANKA for 4-5 hours. After stimulation, the cells were collected for intracellular staining.

**Supplementary Figures**

**
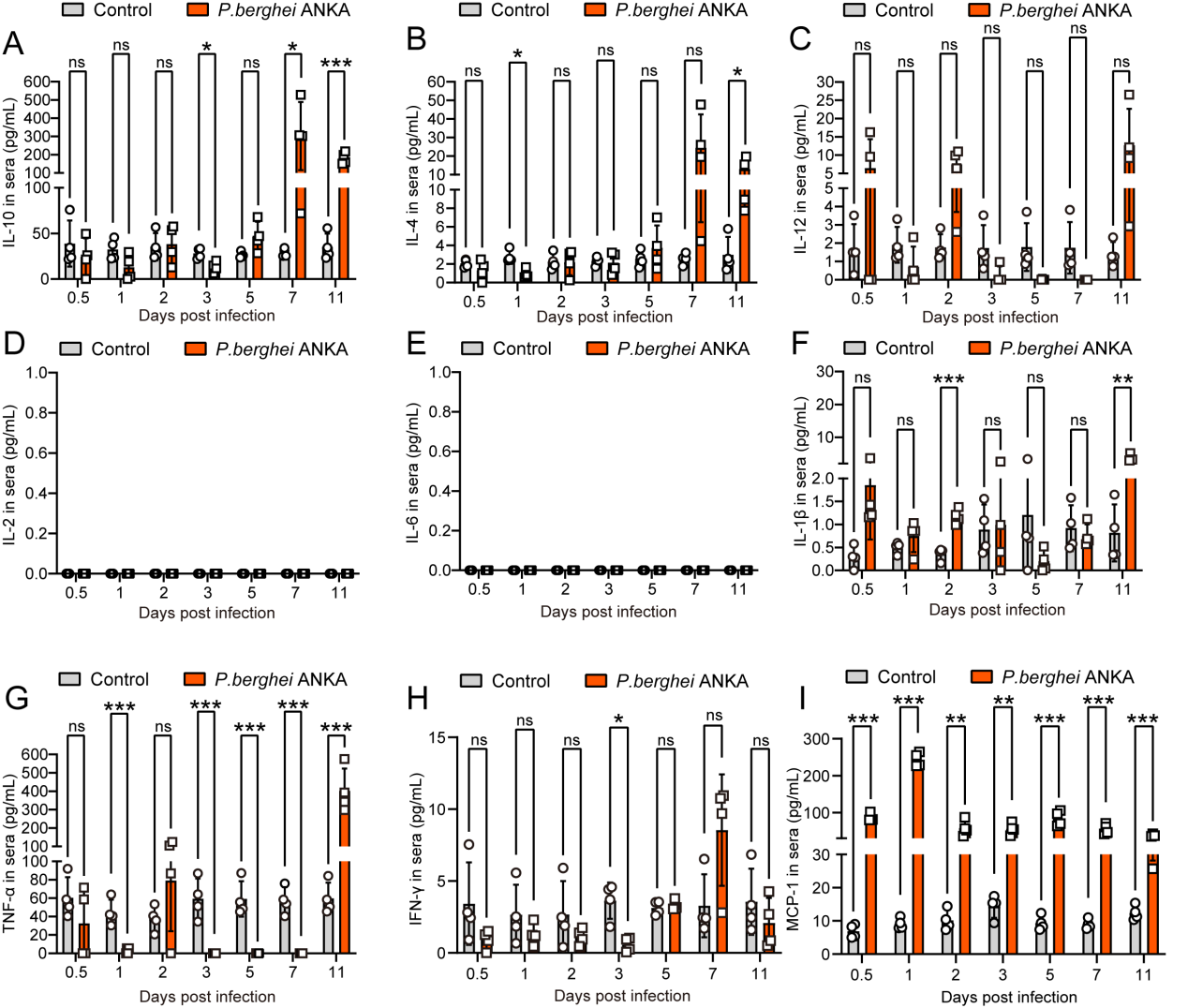
**

**Fig. S1. Responses of various cytokines at different time points post *P. berghei* ANKA infection.** (A and B) The levels IL-10 and IL-4 increased significantly on day 7 and 11 (n=4). (C) The levels of IL-12 showed no significant changes after infection (n=4). (D and E) No responses of IL-2 and IL-6 were observed after infection (n=4). (F) The levels of IL-1β increased significantly on days 2 and 11 after infection (n=4). (G) The levels of TNF-α decreased significantly on days 1, 3, 5 and 7, and increased significantly on day 11. (n=4). (H) The levels of IFN-γ decreased significantly on day 3 (n=4). (I) The levels of MCP-1 increased significantly after infection (n=4). ns, *p*>0.05; *, *p*<0.05; **, *p*<0.01; ***, *p*<0.001.


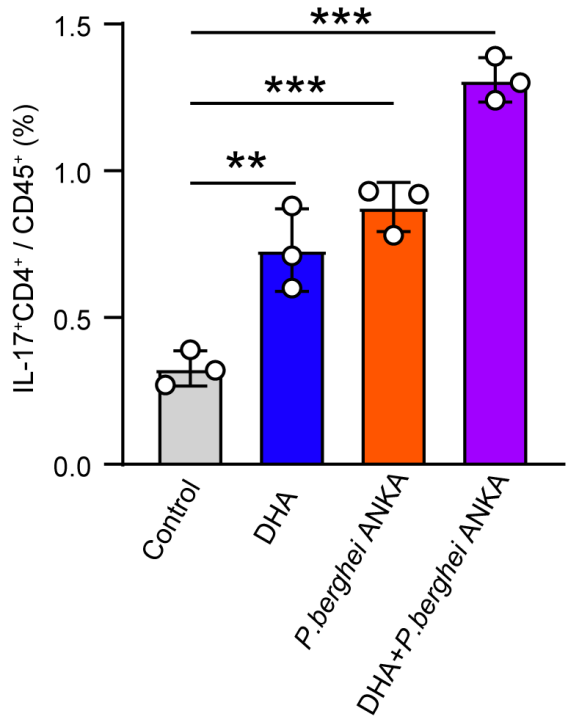


**Fig. S2. DHA promoted the differentiation of CD4^+^ T cells into Th17 cells during *P. berghei* ANKA infection.** After stimulating CD4^+^ T cells with DHA and *P. berghei* ANKA, the proportion of IL-17^+^ cells were significantly increased (*p*=0.0105 and *p*=00008), but the proportion of IL-17^+^ cells was higher in the presence of both DHA and *P. berghei* ANKA (*p*<0.0001). ns, *p*>0.05; *, *p*<0.05; **, *p*<0.01; ***, *p*<0.001.


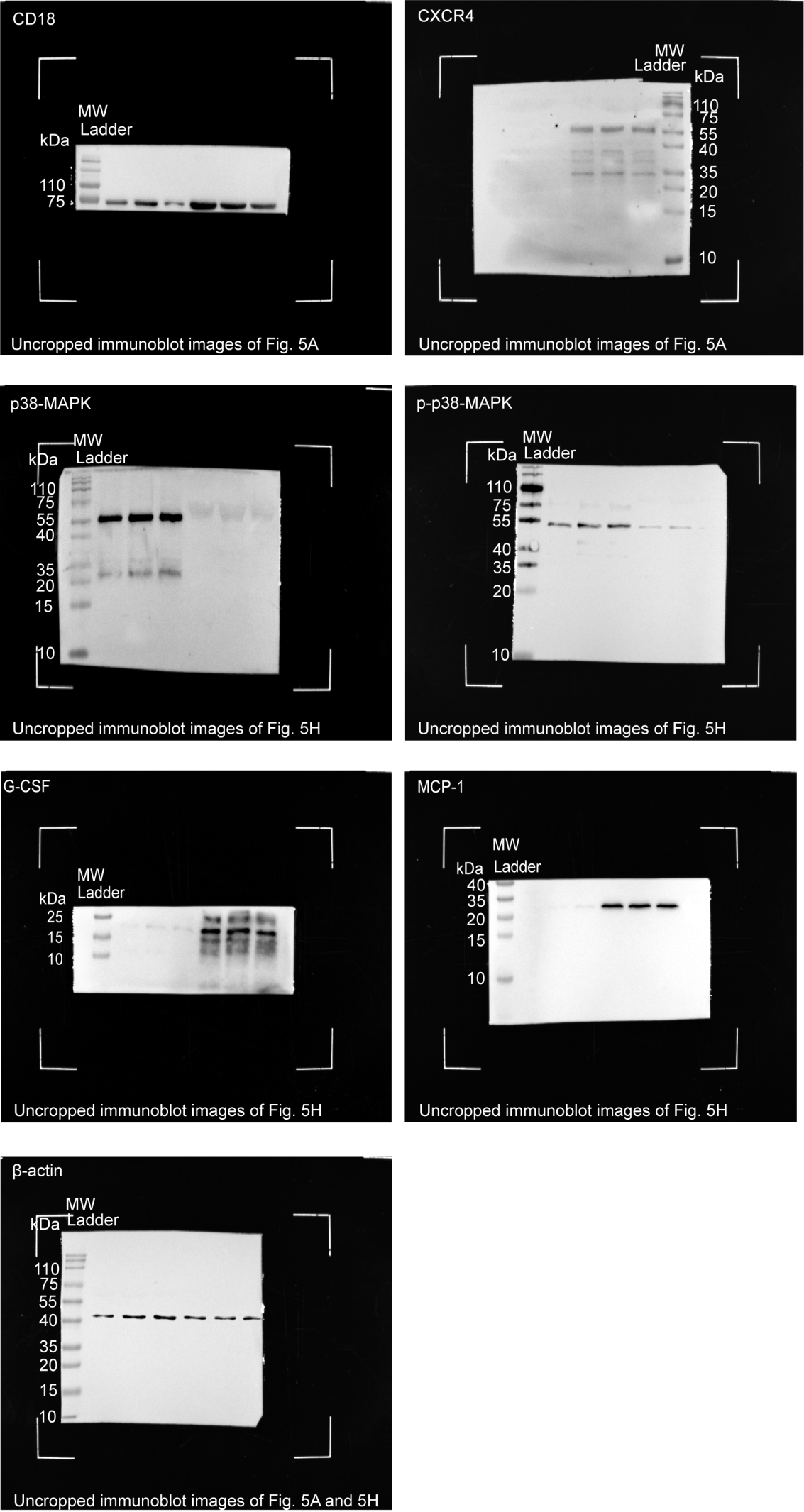


**Fig. S3. Uncropped immunoblot images.**


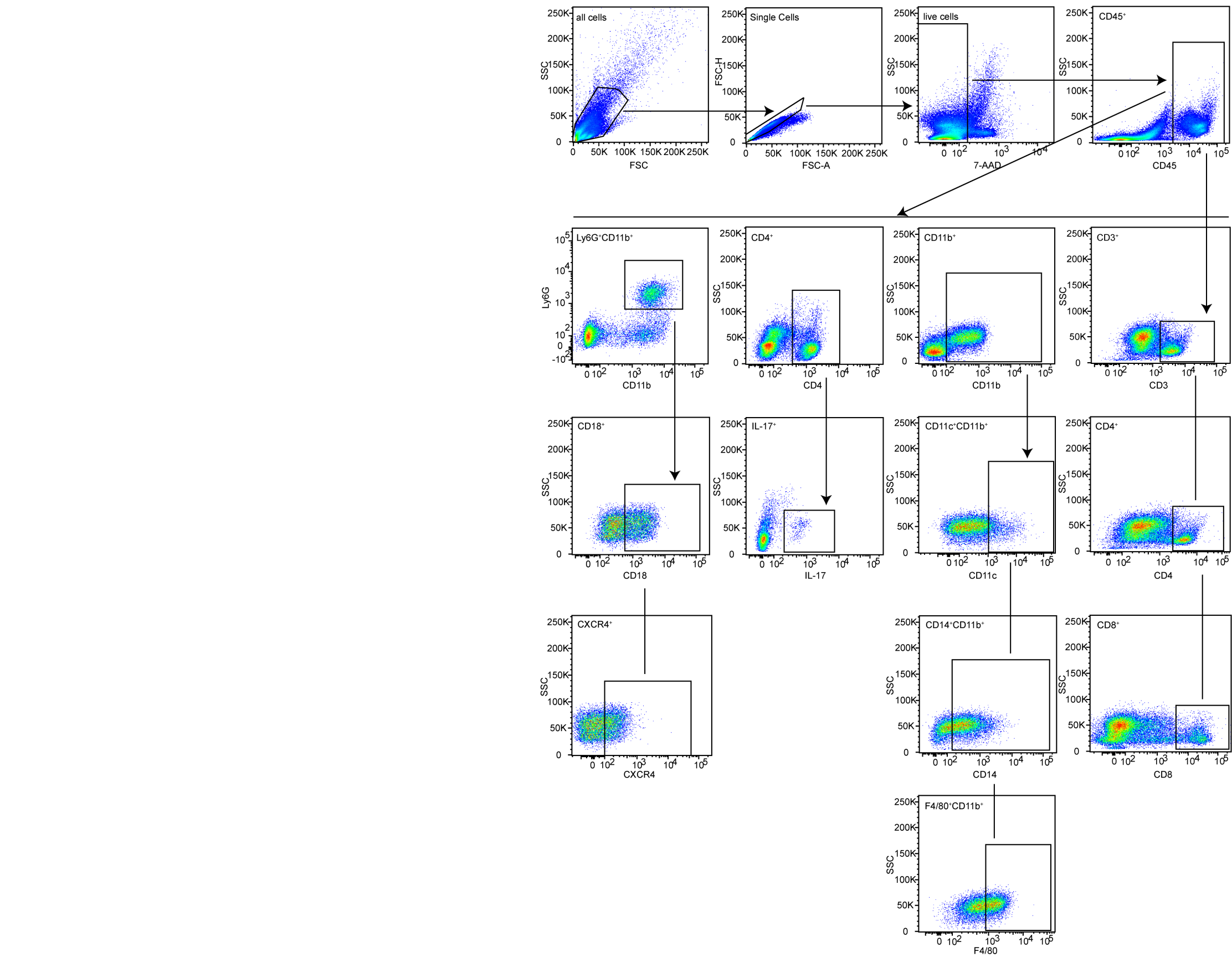


**Fig. S4. Gating strategy.**

**Supplementary Table**

**Table S1. Key antibodies for the present research**

| Reagent | Dosage | concentration | Clone | Cat | Brands |
| --- | --- | --- | --- | --- | --- |
| anti-Ly6G | 200 µg | 7.1 mg/mL | 1A8 | L280 | Leinco |
| Rat IgG2a | 200 µg | 7.1 mg/mL | 1-1 | I-1177 | Leinco |
| anti-IL-17 | 200 µg | 5 mg/mL | 17F3 | BP0173 | BioXcell |
| Mouse IgG1 | 200 µg | 5 mg/mL | MOPC-21 | BE0083 | BioXcell |
| TruStain FcX™ (anti-mouse CD16/32) Antibody | 1 µg | 0.5 mg/mL | 93 | 101320 | Biolegend |
| PE/Cyanine7 Anti-Mouse CD45 Antibody | 5 µL | 500 µL | 30-F11 | E-AB-F1136H | Elabscience |
| APC Anti-Mouse/Human CD11b Antibody | 5 µL | 500 µL | M1/70 | E-AB-F1081E | Elabscience |
| PE Anti-Mouse Ly6G Antibody | 5 µL | 500 µL | 1A8 | E-AB-F1108D | Elabscience |
| FITC Anti-Mouse Ly6G Antibody | 5 µL | 500 µL | 1A8 | E-AB-F1108C | Elabscience |
| FITC Anti-Mouse CD3 Antibody | 5 µL | 500 µL | 17A2 | E-AB-F1013C | Elabscience |
| PE Anti-Mouse CD4 Antibody | 5 µL | 500 µL | RM4-5 | E-AB-F1353D | Elabscience |
| APC Anti-Mouse CD8a Antibody | 5 µL | 500 µL | 53-6.7 | E-AB-F1104E | Elabscience |
| FITC Anti-Mouse F4/80 Antibody | 5 µL | 500 µL | CI: A3-1 | E-AB-F0995C | Elabscience |
| PE Anti-Mouse CD14 Antibody | 5 µL | 500 µL | Sa14-2 | E-AB-F1176D | Elabscience |
| Elab Fluor Violet 450 Anti-Mouse CD11c Antibody | 5 µL | 500 µL | N418 | E-AB-F0991Q | Elabscience |
| FITC Anti-Mouse CD4 Antibody | 5 µL | 500 µL | RM4-5 | E-AB-F1353C | Elabscience |
| PE Anti-Mouse IL-17A Antibody | 5 µL | 500 µL | TC11-18H10.1 | E-AB-F1199D | Elabscience |
| PE anti-mouse CD18 Antibody | 1 µg | 0.2 mg/mL | M18/2 | 101407 | Biolegend |
| Brilliant Violet 605™ anti-mouse CD184 (CXCR4) Antibody | 0.25 µg | 0.2 mg/mL | L276F12 | 146519 | Biolegend |
| ITGB2/CD18 Antibody | 1:1000 | 50 µL | P05107 | DF6896 | Affinity |
| CXCR4 Mouse Monoclonal Antibody | 1:1000 | 100 µL | P61073 | BF8402 | Affinity |
| MCP1 Antibody | 1:2000 | 50 µL | P13500 | DF7577 | Affinity |
| G-CSF Antibody | 1:2000 | 50 µL | EPR3203 | ab181053 | abcam |
| Phospho-p38 MAPK (Thr180/Tyr182) Antibody | 1:1000 | 50 µL | Q16539 | AF4001 | Affinity |
| p38 MAPK Monoclonal Antibody | 1:5000 | 100 µL | Q16539 | BF8015 | Affinity |
| beta Actin Antibody | 1:10000 | 50 µL | P60709 | AF7018 | Affinity |
